# Small Interfering RNA against Transcription Factor STAT6 Leads to Increased Cholesterol Synthesis in Lung Cancer Cell Lines

**DOI:** 10.1371/journal.pone.0028509

**Published:** 2011-12-05

**Authors:** Richa Dubey, Ravindresh Chhabra, Neeru Saini

**Affiliations:** Functional Genomics Unit, Institute of Genomics and Integrative Biology (CSIR), Delhi, India; South Texas Veterans Health Care System, United States of America

## Abstract

STAT6 transcription factor has become a potential molecule for therapeutic intervention because it regulates broad range of cellular processes in a large variety of cell types. Although some target genes and interacting partners of STAT6 have been identified, its exact mechanism of action needs to be elucidated. In this study, we sought to further characterize the molecular interactions, networks, and functions of STAT6 by profiling the mRNA expression of STAT6 silenced human lung cells (NCI-H460) using microarrays. Our analysis revealed 273 differentially expressed genes after STAT6 silencing. Analysis of the gene expression data with Ingenuity Pathway Analysis (IPA) software revealed *Gene expression, Cell death, Lipid metabolism* as the functions associated with highest rated network. *Cholesterol biosynthesis was* among the most enriched pathways in IPA as well as in PANTHER analysis. These results have been validated by real-time PCR and cholesterol assay using scrambled siRNA as a negative control. Similar findings were also observed with human type II pulmonary alveolar epithelial cells, A549. In the present study we have, for the first time, shown the inverse relationship of STAT6 with the cholesterol biosynthesis in lung cancer cells. The present findings are potentially significant to advance the understanding and design of therapeutics for the pathological conditions where both STAT6 and cholesterol biosynthesis are implicated viz. asthma, atherosclerosis etc.

## Introduction

STAT6 is one of the seven members of the family of transcription factors that participate in the regulation of gene expression when cells encounter various extracellular polypeptides like cytokines, hormones and growth factors and regulate a broad range of cellular processes including proliferation, differentiation and apoptosis [1], [2], [3], [4]. In general, unphosphorylated STAT proteins exist as latent forms in the cytoplasm. The cytokine exposure leads to STAT phosphorylation by Janus kinases and once phosphorylated the dimerization of individual STAT proteins occur via their SH2 domains followed by migration of functional STAT dimer to the nucleus where it can bind DNA and directly activate transcription of cytokine responsive genes [5], [6]. Just like the other members of the STAT family, STAT6 plays a dual role of signal transducer and activator of transcription by either directly regulating gene expression or by interacting with a wide variety of other transcription factors [7].

IL-4 and IL-13 induced STAT6 signaling has been shown to play an important role in the differentiation of Th2 cells, B cell induced expression of IgG and IgE and the cell surface display of MHC class II and CD23 [8], [9], [10], [11]. Though STAT6 is primarily known to be associated with allergic inflammation and asthma, STAT6 deregulation has also been implicated in various other diseases. STAT6 plays a key role in T cell hepatitis via enhancing expression of eotaxins in hepatocytes and endothelial cells, and induces IL-5 expression, infiltration of eosinophils and neutrophils into the liver and leading to hepatitis [12]. There are also evidences that IL-4-induced activation of STAT6 is associated with reduced hepatic expression of TNFα as well as attenuation of liver neutrophil recruitment and may protect against hepatic ischemia/reperfusion injury [13]. STAT6 has also been demonstrated to be involved in ciliary mechanosensation in kidney epithelial cell [14]. Recently, IL-4 and STAT6 gene polymorphisms have also been found associated with systemic lupus erythematosus development in Chinese patients [15]. Shum *et al* in 2006 provided a link between allergic inflammation and fatty acid metabolism where they have shown that an IL-4/STAT6 regulated gene aP2, which plays an important role in lipid metabolism, is required in Th2 mediated allergic airway inflammation [16] and recently STAT6 has been found to play a role in regulating lipid homeostasis in liver as increased lipid deposition was observed in STAT6 knockout mice [17]. In addition to the above findings, Zhang *et al* in 2006 reported that STAT6 silencing inhibits proliferation and induces apoptosis in colon cancer HT-29 cells [4]. In another study, Das *et al* in 2007 found that STAT6 is a constitutively expressed survival factor in human prostate cancer [18]. This effect of STAT6 was further strengthened in a study by Cui *et al* in 2007, where they have shown that unphosphorylated STAT6 transcriptionally up regulates COX-2 expression and protects against apoptosis in NSCLC (non-small cell lung cancer) cells [19].

Although, a few target genes and some interacting partners of STAT6 have been known till date, the precise mechanisms of STAT6 mediated signaling is largely unknown. In view of this, we sought to study the effect of STAT6 silencing on genome wide gene expression patterns in NCI-H460 cells (lung cancer epithelial). The results obtained after siRNA mediated silencing of STAT6 in NCI-H460 cells were also validated in A549 cells.

## Materials and Methods

### Cell culture and siRNA Transfection

Lung carcinoma (NCI-H460 and A549) cells were obtained from National Centre for Cell Science, Pune, India and maintained in RPMI-1640/DMEM media, containing 10% fetal calf serum and antibiotics (100 U/ml penicillin, 100 µg/ml streptomycin) at 37°C in a humidified atmosphere of 5% CO_2_ in air.

For transfection in 12 well plates, 1.2×10^5^ cells were seeded per well and allowed to adhere overnight. The following day cells were transfected with 60 nM of validated siRNA (Ambion, USA) using 4 µl of lipofectamine 2000 (Invitrogen, USA) according to the manufacturer's protocol. Wherever indicated, cells were stimulated with 50 ng/ml recombinant human IL-4 (BD Pharmingen, USA) for 4 h. The cells were harvested after 24 h/48 h/72h post transfection and used for the experiments. The untransfected and scrambled siRNA transfected cells were harvested after 48 h for all the experiments unless otherwise indicated.

### RNA Extraction and Real Time PCR

Total RNA was extracted using Trizol reagent (Invitrogen, CA, USA) and 2 µg of RNA was reverse transcribed using RevertAid™ H Minus Reverse Transcriptase kit (Fermentas, USA) according to the manufacturer's protocol**.** Real time PCR was done using SYBR Green PCR master mix (Applied Biosystems, Foster City, CA). Results were normalized with 18s rRNA. Data was analysed using Pfaffl's method [20]. The primer sequences used for RT-PCR are given in Table S1.

### Western Blotting

Cells were trypsinized and cell pellets were lysed with modified RIPA buffer {50 mM Tris-HCl, pH 7.4, 150 mM NaCl, 1 mM EDTA, 1% NP40, 0.25% Na deoxycholate, 1 µg/ml aprotinin, 1 µg/ml leupeptin, 1 µg/ml pepstatin, 1 mM phenylmethylsulfonyl fluoride (PMSF), 1 mM sodium orthovandate, and 1mM sodium fluoride} and kept in ice for 30 min. Lysate was centrifuged at 12000 rpm for 30 min, supernatant collected and protein estimation was done using the BCA method. Equal amounts of protein (50 µg) were separated on 12% sodium dodecyl sulphate – polyacrylamide gel electrophoresis (SDS-PAGE) and transferred to PVDF membrane. The membrane was blocked with 3% skim milk in Tris buffered saline (20 mM Tris, 150 mM NaCl, pH 7.4) with 0.1% Tween-20 for 1 h and then incubated with primary antibody in 1% skim milk for 2 h followed by incubation with appropriate secondary antibody (anti-mouse ALP linked or/anti-rabbit ALP linked) for 1 h. Blots were developed using NBT- BCIP as substrate. Equal loading of protein was confirmed using GAPDH antibody. Measurement of signal intensity on PVDF membranes after western blotting was performed using AlphaImager 3400 (Alpha InnoTech Corporation, San Leandro, California). The IDV values are calculated as the density values of the specific protein band/GAPDH density values. The fold change with respect to untransfected cells was then calculated based on IDV values. All experiments were repeated at least three times; representative results are presented.

### Illumina Microarray

Genome wide effect of STAT6 silencing was studied using Illumina microarray. Two biological replicates of untransfected NCI-H460 cells and NCI-H460 cells transfected with 60nM of STAT6 siRNA (for 48 h) were used in the array experiment. Total RNA was extracted using Trizol reagent (Invitrogen, CA, USA), purified and concentrated using RNeasy MinElute Cleanup Kit (Qiagen, CA, USA) as per the manufacturer's protocol. All RNA samples were tested for integrity by gel electrophoresis. The Illumina TotalPrep RNA Amplification Kit (Ambion, TX, USA) was used to generate biotinylated, amplified RNA. In brief, 500 ng of total RNA was reverse transcribed with an oligo(dT) primer using ArrayScript enzyme and amplified overnight with T7 RNA polymerase and labeled with biotin according to the manufacturer's protocol. This labelled amplified RNA (aRNA) was hybridized to Illumina Genome-Wide Expression BeadChips (Human Ref-6 v.3.0, Illumina, CA, USA) representing ∼43,000 human transcripts at 58°C overnight. Arrays were incubated with Cy3 streptavidin and washed according to the manufacturer's protocol. The chip was scanned using Illumina scanner (iScan) and the analysis of the microarray data was done using Illumina Beadstudio 2.0 software. The data was average normalized and the genes which crossed the threshold of detection p value ≤ 0.05 among all the samples and differential score p value ≤ 0.05 among the test samples were considered to be differentially expressed genes. The work-flow diagram of this experiment is given in Figure S1. The data obtained has been deposited in NCBI's Gene Expression Omnibus [21] and is accessible through Gene Expression Omnibus (GEO) Series accession number GSE25942 (http://www.ncbi.nlm.nih.gov/geo/query/acc.cgi?acc=GSE25942).

### Ingenuity pathway analysis (IPA)

Datasets representing genes with altered expression profile derived from microarray analyses were imported into the Ingenuity Pathway Analysis Tool (IPA Tool; Ingenuity®Systems, Redwood City, CA, USA; http://www.ingenuity.com). In IPA, differentially expressed genes are mapped to genetic networks available in the Ingenuity database and then ranked by score.

The basis of the IPA program consists of the Ingenuity Pathway Knowledge Base (IPKB) which is derived from known functions and interactions of genes published in the literature. Thus, the IPA Tool allows the identification of biological networks, global functions and functional pathways of a particular dataset. The program also gives the significance value of the genes, the other genes with which it interacts, and how the products of the genes directly or indirectly act on each other, including those not involved in the microarray analysis. The networks created are ranked depending on the number of significantly expressed genes they contain and also list diseases that were most significant. A network is a graphical representation of the molecular relationships between molecules. Molecules are represented as nodes, and the biological relationship between two nodes is represented as an edge (line). All edges are supported by at least 1 reference from the literature, from a textbook, or from canonical information stored in the Ingenuity Pathways Knowledge Base. The intensity of the node color indicates the degree of up- (red) or down- (green) regulation. Nodes are displayed using various shapes that represent the functional class of the gene product.

### PANTHER analysis

The PANTHER (**P**rotein **AN**alysis **TH**rough **E**volutionary **R**elationships) Classification System is a unique resource that classifies genes by their functions, using published scientific experimental evidence and evolutionary relationships to predict function even in the absence of direct experimental evidence [22]. The differentially expressed genes obtained after STAT6 silencing in NCI-H460 cells were imported into PANTHER (http://www.pantherdb.org/), where the number of genes in each pathway were compared against the number of genes from NCBI's *Homo sapiens* genome in that pathway. The binomial test was used to statistically determine overrepresentation of PANTHER classification categories. Bonferroni-corrected p values < 0.05 were considered significant.

### Cholesterol assay

Cholesterol content was determined in the cells using cholesterol quantitation kit (Biovision, CA, USA) according to the manufacturer's instruction. In brief, 10^6^ cells were lysed and lipids were extracted by homogenization with 200 µl of chloroform: isopropanol: Triton X-100 (7:11:0.1). These lipid extracts were vacuum dried for 30 min and the residues were dissolved in 200 µl cholesterol Reaction Buffer provided with the kit. Cholesterol was estimated by spectrophotometry at λ = 570 nm in a 96 well plate according to the manufacturer's instructions. The cholesterol levels were normalized to amounts of total cellular protein.

### Promoter Analysis

To identify the common regulatory controls among the altered genes, genes of the cholesterol biosynthesis pathway (HMGCR- 3-hydroxy-3-methylglutaryl-Coenzyme A reductase, HMGCS1- 3-hydroxy-3-methylglutaryl-Coenzyme A synthase 1 and IDI1- Isopentenyl-diphosphate delta isomerase 1) were taken for promoter analysis. Ensembl [23] was used to retrieve 5.0 kb upstream regions from the transcription start sites of these genes and the transcription factor(s) binding within this region was determined using Over-represented Transcription Factor Binding Site Prediction (OTFBS) tool [24] that detects over-represented motifs of known transcription factors for a set of genes.

### Electrophoretic Mobility Shift Assay (EMSA)

The electrophoretic mobility shift assay was carried out as described by Shiraga *et al*. [37]. Nuclear extracts were prepared from untransfected, siRNA-transfected (60 nM, 48 h/72 h) and scrambled siRNA transfected NCI-H460 cells by using NE-PER Nuclear and Cytoplasmic Extraction Reagent Kit (Pierce) according to the manufacturer's protocol.

The oligonucleotide sequences used in EMSA were taken from an earlier published study [25]. Double stranded DNA was generated by mixing equimolar amounts of the complementary oligonucleotides in annealing buffer (Ambion, CA, USA). Mutated sequence of the transcription factor was also used to check the specificity of the binding of the transcription factor. These annealed double stranded DNA were labeled with [c32-P]-ATP (BRIT, Hyderabad, India) in the presence of T4-polynucleotidekinase (New England Biolabs, MA, USA) according to manufacturer's instructions. Nuclear extracts (18 µg) from the untransfected and siRNA-transfected cells were incubated for 30 min at room temperature in the presence of reaction buffer containing 8 mM Tris-KCl (pH 8.0), 2 mM EDTA, 1 mM DTT, 12% glycerol, 1 mg BSA and 1 mg of poly(dI-dC). Either the wild type or mutated labeled double stranded oligonucleotide (40,000 cpm) was then added to the reaction mixture and incubated for 45 min at room temperature. On termination of incubation, samples were loaded onto a non-reducing 6% polyacrylamide gel and electrophoresed in 0.5X TBE. Gels were then dried and subjected to phosphorimager analyses (FLA 2000, Fujifilm, Japan). The densitometric analyses were done using the AlphaImager 3400 (Alpha InnoTech Corporation, San Leandro, California). The same size rectangle box was drawn surrounding each band and the intensity of each was analyzed by the program after subtraction of the background intensity.

### Annexin-V assay

Apoptosis was assessed by the Guava Nexin kit and the Guava PCA system (Guava Technologies, Hayward, CA, USA). The exposure of phosphatidyl serine (PS) on the cell surface (associated with the onset of apoptosis) forms the basis of the Guava Nexin assay. The Guava Nexin assay utilizes two stains (annexin V and 7-amino actinomycin D [7-AAD]). Annexin V-PE binds to PS on the cell surface of apoptotic cells and 7-AAD, the cell impermeant dye is an indicator of membrane structural integrity. 7-AAD is excluded from live, healthy and early apoptotic cells, but permeates late stage apoptotic and dead cells. The assay was performed according to the manufacturer's protocol and Annexin-PE fluorescence was analyzed with the help of cytosoft software (Guava Technologies, Hayward, CA, USA). A minimum of 2,000 events were counted.

### Cell cycle assay

For analysis of cell cycle distribution, cells were fixed with ice-cold 70% ethanol and treated with 1mg/ml RNase for 30 minutes at 37°C. The cells were then treated with fluorescence dye propidium iodide (50 µg/ml, Sigma, USA) which bind to DNA by intercalating between the bases at 4°C for 30 minutes and analyzed using flow cytometer (Guava Technologies, Hayward, California, USA). A minimum of 5,000 events were counted.

## Results

### STAT6 downregulation using STAT6 specific siRNA

**Figure 1:**
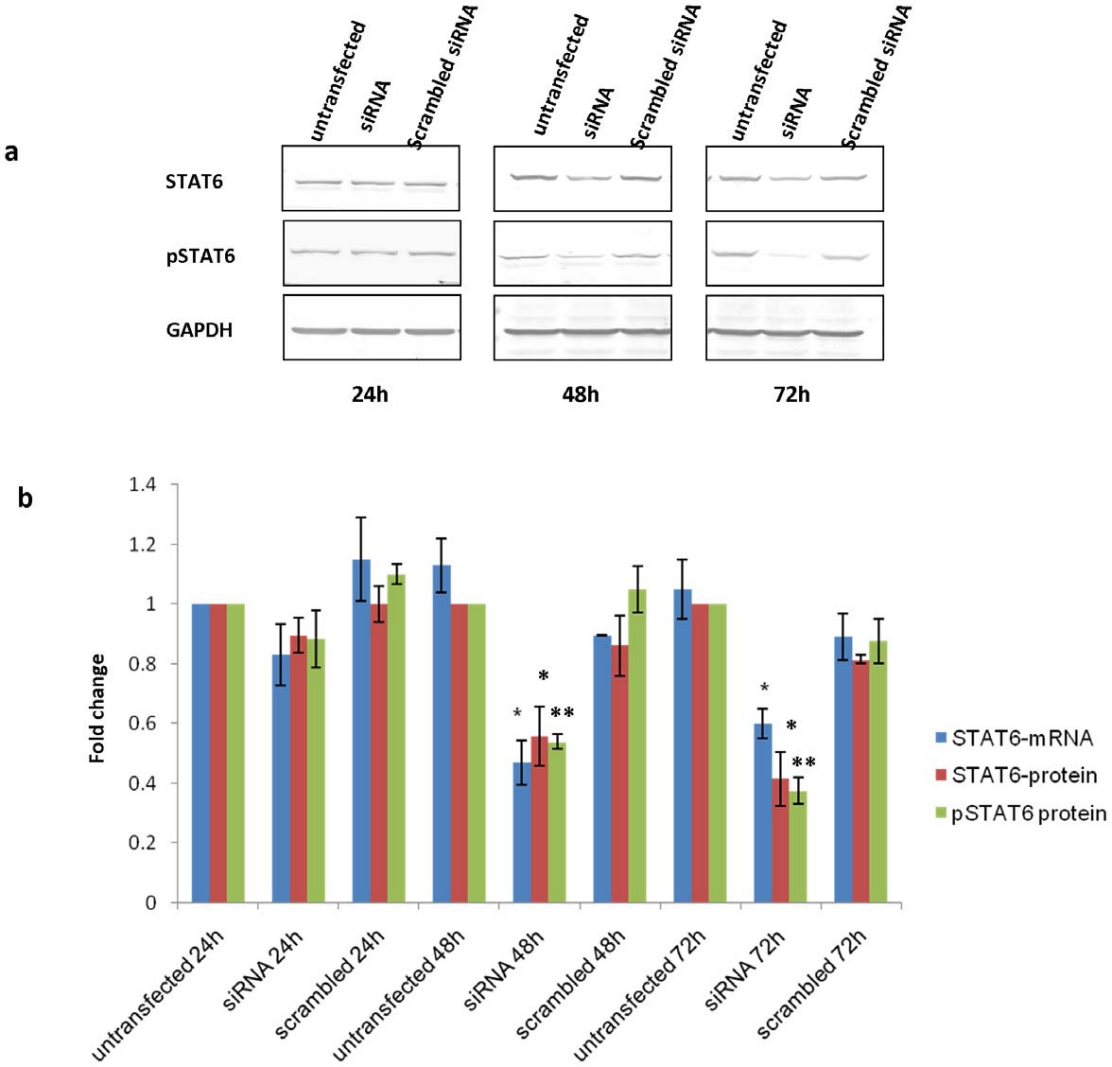
Silencing of STAT6 in NCI-H460 cells using STAT6 specific siRNA. a) The western blot of STAT6 and phosphorylated form of STAT6 (pSTAT6) protein on cell extracts from untransfected, STAT6 specific siRNA and scrambled siRNA transfected NCI-H460 cells at different time points as indicated. b) Graph represents the fold change in STAT6 mRNA level, STAT6 protein and pSTAT6 protein level compared to untransfected NCI-H460 cells at respective time points. Scrambled siRNA at different time points was used as a negative control. GAPDH was used as a loading control for densitometric analysis for western blot analysis. The mRNA levels were normalized to 18s rRNA expression in Real time PCR analysis. The data is expressed as the mean ± S.D. of 3 independent experiments. * indicates p value < 0.05 in comparison to untransfected cells. ** indicates p value < 0.01 in comparison to untransfected cells. c).

The efficacy of STAT6 specific siRNA to down regulate STAT6 expression in NCI-H460 cells was evaluated by real time PCR for RNA expression and western blotting for protein levels. As shown in Fig. 1b **,** the RNA levels decreased by 1.20 fold at 24h, 2.12 fold (p value = 0.046) at 48 h and by 1.66 fold (p value  =  0.05) at 72 h in siRNA transfected NCI-H460 cells in comparison to untransfected NCI-H460 cells. There was no significant change in the untransfected cells and scrambled siRNA transfected cells at different time points. However, the protein levels of STAT6 were reduced in a time-dependent manner. As shown in Fig. 1a and 1b, there was 1.12 fold decrease at 24 h, 1.79 fold (p value  =  0.02) decrease at 48 h and 2.40 fold (p value  =  0.028) decrease at 72 h post transfection of STAT6 specific siRNA in NCI-H460 cells in comparison to untransfected NCI-H460 cells at respective time points. We next checked the expression of phosphorylated STAT6 (pSTAT6) protein which is the activated signaling form of STAT6. In concordance with STAT6 protein levels, the expression of pSTAT6 protein levels was also reduced in a time-dependent manner. There was 1.13 fold decrease at 24 h, 1.85 fold (p value  =  0.0008) decrease at 48 h and 2.67 fold (p value  =  0.001) decrease at 72 h post transfection of STAT6 specific siRNA in NCI-H460 cells when compared with untransfected NCI-H460 cells at respective time points (Fig. 1a and b). Non significant changes were observed in cells transfected with scrambled siRNA (negative control) at different time points.

### Genome wide effects of STAT6 silencing in NCI-H460 cell line using Illumina microarray

The gene expression profiles in untransfected NCI-H460 cells and NCI-H460 cells transfected with STAT6 siRNA were determined by illumina microarray using two biological replicates. Illumina array experiment identified 273 differentially expressed genes (*187 downregulated and 86 upregulated)*. Raw data was analysed using Beadstudio 2.0 software. The array data was average normalized and filtered by detection of a p value ≤ 0.05 and differential p value ≤ 0.05. The list of differentially expressed genes along with their fold changes is provided in the Table S2.

### Elucidation of pathways and interactions among differentially expressed genes

To investigate possible biological interactions of differently regulated genes, datasets representing genes with altered expression profile derived from microarray analyses were imported into the Ingenuity Pathway Analysis Tool.

**Figure 2:**
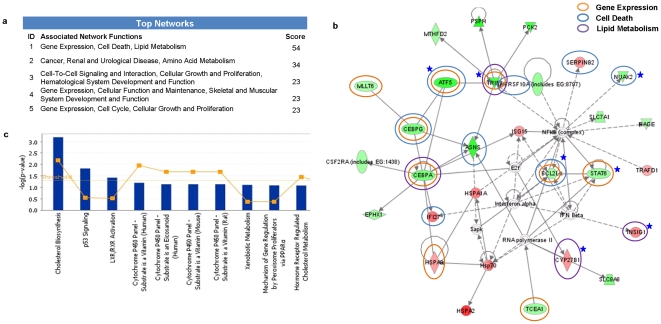
Ingenuity Pathways Analysis (IPA) summary. To investigate possible interactions of differently regulated genes, datasets representing 273 genes with altered expression profile obtained from the illumina microarray were imported into the Ingenuity Pathway Analysis Tool and the following data is illustrated: a) The list of top five networks with their respective scores obtained from IPA b) Most highly rated network in IPA analysis The network representation of the most highly rated network (Gene Expression, Cell Death, Lipid Metabolism). The genes that are shaded were determined to be significant from the statistical analysis. The genes shaded red are upregulated and those that are green are downregulated. The intensity of the shading shows to what degree each gene was up or downregulated. A solid line represents a direct interaction between the two gene products and a dotted line means there is an indirect interaction. The genes marked with blue asterisk have been validated by real time PCR. Genes associated with Gene expression, Cell Death and Lipid Metabolism are circled with orange, blue and purple colours, respectively. c) Toxicology pathway list in IPA analysis. The x-axis represents the top toxicology functions as calculated by IPA based on differentially expressed genes are highlighted and the y-axis represents the ratio of number of genes from the dataset that map to the pathway and the number of all known genes ascribed to the pathway. The yellow line represents the threshold of p value < 0.05 as calculated by Fischer's test.

The list of differentially expressed genes analyzed by IPA revealed 20 significant networks (Table S3). Fig. 2a represents the list of top 5 networks identified by IPA. Of these networks, *Gene expression, Cell death, Lipid metabolism* was the highest rated network with 28 focus molecules and the significance score of 54 (Fig. 2b). The score is the probability that a collection of genes equal to or greater than the number in a network could be achieved by chance alone. A score of 3 indicates a 1/1000 chance that the focus genes are in a network not due to random chance. The list of genes in this network with their respective fold changes in the array data is provided in the Table S4.

The IPA analysis also groups the differentially expressed genes into biological mechanisms that are related to toxicity groups. In the toxicology list, *Cholesterol biosynthesis and p53 Signaling* came out to be the top two most significant pathways with a p value of 0.011 and 0.013, respectively (Fig. 2c). The genes associated with the top tox list are also given in the Table S5.

Simultaneously, differentially expressed gene list obtained after STAT6 silencing in NCI-H460 cells was fed into the PANTHER web resource to reveal enriched pathways. Interestingly, in this analysis too we found cholesterol biosynthesis and apoptosis signaling among the significantly enriched pathways. The pathways which passed the threshold of p value < 0.05 in PANTHER analysis are listed in Table 1.

**Table 1:** Enriched pathways from PANTHER analysis^a^.

Pathways	Homo sapiens genes (reference)	Differentially expressed gene list			
	#	#	expected	+/-	p value
Cholesterol biosynthesis	13	3	0.16	+	5.71E-04
Serine glycine biosynthesis	5	2	0.06	+	1.75E-03
Apoptosis signaling pathway	123	6	1.49	+	4.15E-03
Oxytocin receptor mediated signaling pathway	60	4	0.73	+	6.42E-03
Thyrotropin-releasing hormone receptor signaling pathway	62	4	0.75	+	7.19E-03
Cytoskeletal regulation by Rho GTPase	98	5	1.19	+	7.22E-03
5HT2 type receptor mediated signaling pathway	69	4	0.84	+	1.03E-02
Cysteine biosynthesis	1	1	0.01	+	1.20E-02
Histamine H1 receptor mediated signaling pathway	47	3	0.57	+	2.00E-02
Lipoate_biosynthesis	2	1	0.02	+	2.39E-02
Proline biosynthesis	4	1	0.05	+	4.73E-02

(**^a^** # - number of genes, expected - the number of genes expected in the list for this PANTHER category, based on the reference list, +/- - Over representation of a category is denoted by a + sign and under representation by a – sign).

### STAT6 silencing increases expression of genes associated with cholesterol biosynthesis/homeostasis and enhances cholesterol levels

**Figure 3:**
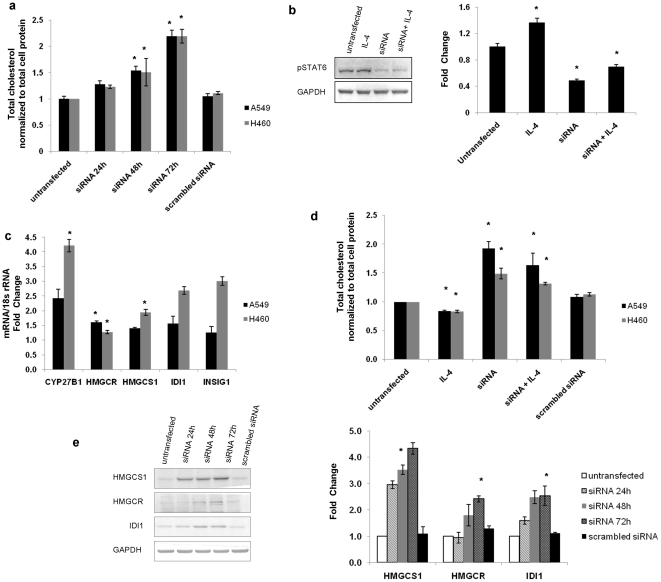
Role of STAT6 in cholesterol biosynthesis. The total cholesterol levels after STAT6 knockdown was checked at different time points in NCI-H460 and A549 cells. Scrambled siRNA was used as negative control. The data is expressed as the mean ± S.D. of 3 independent experiments performed in triplicates. * indicates p value < 0.05 in comparison to untransfected cells. a) Western blot analysis was done to analyze the change in expression of pSTAT6 protein upon IL-4 treatment in NCI-H460 cells. GAPDH was used as a loading control for densitometric analysis. Graph represents the fold change in protein expression compared to untransfected NCI-H460 cells. The data is expressed as the mean ± S.D. of 3 independent experiments. * indicates p value < 0.05 in comparison to untransfected cells. b) Real Time PCR was performed to determine the changes in expression level of genes related to cholesterol biosynthesis and homeostasis in NCI-H460 cells and A549 cells 48h post transfection of STAT6 specific siRNA. The samples were normalized to 18s rRNA expression. The real time data is expressed as the mean ± S.D. of 3 independent experiments performed in triplicates. * indicates p value < 0.05 in comparison to untransfected cells. c) Cholesterol assay was carried out to determine the effect of IL-4 treatment on total cholesterol levels in untransfected and siRNA transfected NCI-H460 cells and A549 cells. The data is expressed as the mean ± S.D. of 3 independent experiments performed in triplicates. * indicates p value < 0.05 in comparison to untransfected cells. d) The western blot of HMGCS1, HMGCR and IDI1 was done on cell extracts from untransfected and siRNA transfected NCI-H460 cells at different time points. GAPDH was used as a loading control for densitometric analysis. Graph represents the fold change in protein expression compared to untransfected NCI-H460 cells. The data is expressed as the mean ± S.D. of 3 independent experiments. * indicates p value < 0.05 in comparison to untransfected cells.

Since the top most network obtained in IPA analysis was *Gene expression, Cell death, Lipid metabolism* and cholesterol biosynthesis came out enriched in both IPA and PANTHER analysis we checked the changes in cholesterol levels after STAT6 silencing by siRNA in NCI-H460 cells. Scrambled siRNA was used as a negative control. Fig. 3a shows that the STAT6 silencing increased cholesterol levels in a time dependent manner, with 1.23 fold increase at 24 h, 1.8 fold (p value  =  0.005) increase at 48 h and 2.3 fold (p value  =  0.004) increase at 72 h post transfection of siRNA in NCI-H460 cells. However, no such change was observed in the cholesterol levels in NCI-H460 cells transfected with scrambled siRNA. We also obtained similar results in A549 cells (Fig. 3a). There was 1.28 fold increase at 24 h, 1.6 fold increase (p value  =  0.03) at 48 h and 2.2 fold increase (p value  =  0.04) at 72 h post transfection of siRNA in A549 cells, thereby indicating that the increase in cholesterol levels could be a general effect of STAT6 silencing on lung cells.

Since IL-4 is known to phosphorylate STAT6, we wanted to look for the role of IL-4 in cholesterol synthesis. In NCI-H460 cells, there was 1.48 fold (p value  =  0.01) change in the level of pSTAT6 (phosphorylated form of STAT6) protein after IL-4 treatment, 0.48 fold (p value  =  0.01) change after transfection of STAT6 siRNA and 0.69 fold (p value  =  0.05) change when the cells transfected with STAT6 siRNA were treated with IL-4 (Fig. 3b). Corresponding to this, there was 0.8 fold (p value  =  0.05) change in the cholesterol levels after IL-4 treatment, 1.5 fold (p value  =  0.01) change after transfection of STAT6 siRNA and 1.32 fold (p value  =  0.03) change when the cells transfected with STAT6 siRNA were treated with IL-4 (Fig. 3d). Similar results were also observed in A549 cell line (Fig. 3d).

We next looked for the differentially expressed genes associated with cholesterol biosynthesis/homeostasis obtained from the microarray data. In concordance with the illumina array data, the transcript levels of these genes were confirmed to be upregulated by real time PCR. We observed 1.27 fold (p value  =  0.03) increase in HMGCR levels, which is the key regulatory enzyme of cholesterol biosynthesis pathway. We also observed 1.94 fold (p value  =  0.03) increase in HMGCS1, 2.7 fold increase in IDI1, 4.2 fold (p value  =  0.04) increase in CYP27B1 *(Cytochrome P450, family 27, subfamily B, polypeptide 1)*, and 3.0 fold increase in INSIG1 *(Insulin induced gene 1)* levels at 48 h post transfection of NCI-H460 cells with STAT6 specific siRNA in comparison to the untransfected NCI-H460 cells (Fig. 3c).

Similar increase in transcript levels of these genes were also observed in another lung cancer cell line, A549 (Fig. 3c) where we observed 1.6 fold (p value  =  0.03) increase in HMGCR levels, 1.4 fold increase in HMGCS1, 1.56 fold increase in IDI1, 2.4 fold increase in CYP27B1, and 1.25 fold increase in INSIG1 levels at 48 h post transfection of A549 cells with STAT6 specific siRNA in comparison to the untransfected A549 cells. This implies that the effects of STAT6 silencing are general and not restricted to any particular lung cancer cell line.

The protein levels of HMGCR, HMGCS1 and IDI1 were also examined in untransfected and siRNA transfected NCI-H460 cells at 48h and 72h post transfection. There were 2.9, 3.5 (p value  =  0.048) and 4.3 fold increase in HMGCS1 levels, 0.94, 1.8 and 2.4 (p value  =  0.03) fold increase in HMGCR levels, 1.6, 2.4 and 2.5 (p value  =  0.024) fold increase in IDI1 levels at 24 h, 48 h and 72 h post transfection, respectively Non significant changes were observed in case of cells transfected with scrambled siRNA (Fig. 3e).

### Promoter analysis and validation of Candidate Transcription Factors

**Figure 4:**
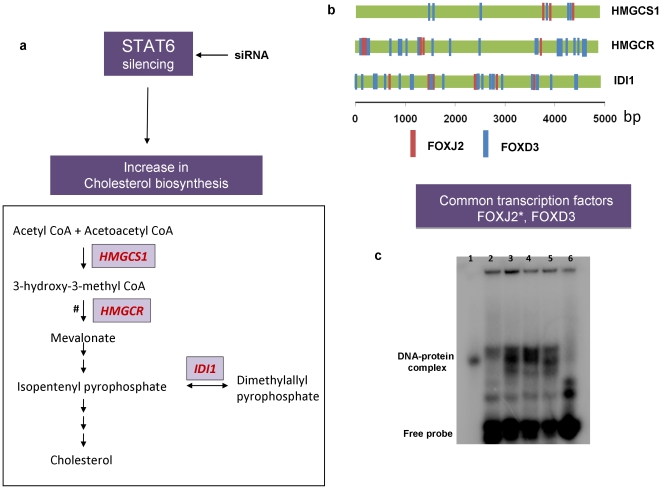
Schematic showing STAT6 silencing effects on cholesterol biosynthesis and validation of FOXJ2 binding by EMSA. a) siRNA mediated STAT6 silencing increases the cholesterol levels of the cell by enhancing the expression levels of enzymes involved in cholesterol biosynthesis pathway. # represents the rate limiting step of the cholesterol biosynthesis pathway. b) The binding sites of FOXJ2 and FOXD3 to the 5kB region upstream of transcription start site of these genes as revealed by OTFBS. 0 bp marks the transcription start site of the gene. c) The transcription factors common to the 3 enzymes of cholesterol biosynthesis pathway that got upregulated after STAT6 silencing were predicted by OTFBS. * transcription factor binding validated by EMSA. 18 μg of nuclear extracts from untransfected or siRNA transfected NCI-H460 cells were incubated with wild type labeled oligonucleotide containing the binding elements of FOXJ2. Mutated probe was used to check the specificity of the DNA-protein complexes. On termination of incubation, samples were resolved in a non-denaturing polyarylamide gel and subjected to phosphorimager analysis. *Lane 1*: labeled FOXJ2 specific oligos, *lane 2*: untransfected NCI-H460 cells incubated with labeled FOXJ2 specific oligos, *lane 3*: NCI-H460 cells tranfected with STAT6 specific siRNA at 48 h incubated with labeled FOXJ2 specific oligos, *lane 4*: NCI-H460 cells tranfected with STAT6 specific siRNA at 72 h incubated with labeled FOXJ2 specific oligos, *lane 5*: NCI-H460 cells tranfected with scrambled siRNA incubated with labeled FOXJ2 specific oligos, *lane 6*: untransfected NCI-H460 cells incubated with labeled mutated FOXJ2 specific oligos.

We next performed the promoter analysis for the 3 enzymes *(HMGCR, HMGCS1, IDI1)* of the cholesterol biosynthesis pathway (Fig. 4a) that are upregulated after STAT6 silencing using the OTFBS, a tool that predicts common transcription factors for a set of genes. This was reasonable as genes with similar functions or that participate in a common process are often transcriptionally coregulated [26], [27]. This analysis revealed FOXD3 and FOXJ2 as the significant potential transcription factors consistently occurring in the promoters of HMGCR, HMGCS1 and IDI1. The binding sites of FOXJ2 and FOXD3 to the 5kB region upstream of transcription start site of these genes as revealed by OTFBS have been shown in Fig. 4b. To validate these transcription factors, electrophoretic mobility shift assay was performed for one of the candidate transcription factors, FOXJ2. As seen from Fig. 4c, when FOXJ2 oligonucleotides were incubated with the nuclear extract of the untransfected NCI-H460 cells, there was considerable formation of the DNA-protein complex in comparison to the free labeled probe. This complex formation increased significantly in case of nuclear extracts from siRNA transfected NCI-H460 cells and this increase was 2.07 fold at 48 h and 2.17 fold at 72 h post transfection of STAT6 specific siRNA indicating that STAT6 silencing increased the binding of FOXJ2 to their binding elements that validate our predicted finding detailed above.

### Effect of STAT6 silencing in NCI-H460 cells on apoptosis and cell cycle

**Figure 5:**
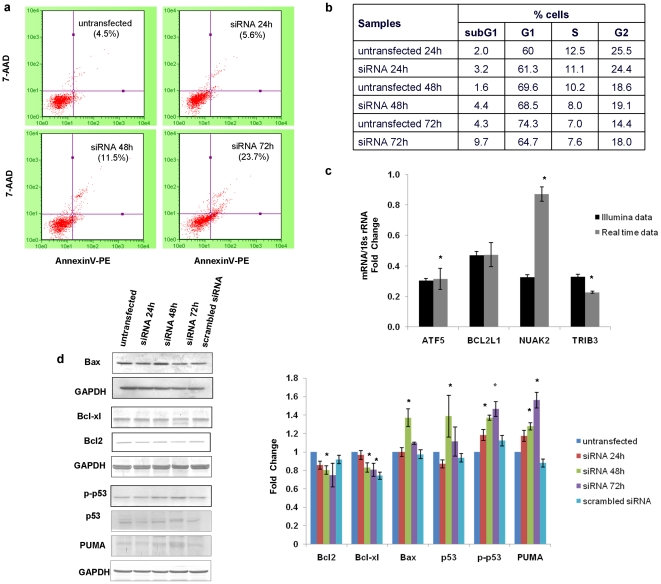
Effect of STAT6 silencing on apoptosis and cell cycle. a) To determine whether STAT6 downregulation in NCI-H460 cells leads to apoptosis, Annexin-V-PE binding assay was done at different time periods as described in “Materials and Methods” section. X-axis represents Annexin V-PE positive cells whereas Y-axis represents 7-AAD positive cells. % here indicates the percentage of dead cells. b) Cell cycle assay was also done to check the distribution pattern of untransfected and siRNA transfected NCI-H460 cells in different phases of cell cycle. Cells were harvested post transfection (at different time points as indicated) and subsequently assayed for their DNA content by flow cytometry. c) Real Time PCR was done in NCI-H460 cells to validate the genes which emerged out to be associated with apoptosis or cell death from the most significant network in the IPA analysis. The samples were normalized to 18s rRNA expression. The real time data is expressed as the mean ± S.D. of 3 independent experiments performed in triplicates. * indicates p value < 0.05 in comparison to untransfected cells. d) Western blot was performed to analyze the expression of some pro-apoptotic and anti-apoptotic proteins in untransfected and siRNA transfected NCI-H460 cells. GAPDH was used as a loading control. The data is expressed as the mean ± S.D. of 3 independent experiments. * indicates p value < 0.05 in comparison to untransfected cells.

Some previous reports have shown the involvement of STAT6 in apoptosis and cell cycle kinetics in different cell types [4], [18], [19]. Since in our study too, *Gene expression, cell death, Lipid metabolism* emerged as the most significant network in IPA analysis and apoptosis signalling pathway as one of the significantly enriched pathways in PANTHER analysis, we thus made an attempt to investigate whether silencing of STAT6 in NCI-H460 has any effect on apoptosis and cell cycle progression. The annexin assay carried out to quantify the number of apoptotic cells (Fig. 5a) revealed that there was a time-dependent increase in annexin V positive cells upon STAT6 silencing in NCI-H460 cells. The percentage of annexin V positive cells increased from 4.5% in untransfected NCI-H460 cells to 5.6% at 24 h, 11.5% (p value  =  0.05) at 48 h and 23.7% (p value  =  0.006) at 72 h post transfection of NCI-H460 cells with STAT6 specific siRNA. We also did the annexin assay in A549 cells but we did not observe any significant changes in the annexin positive cells after STAT6 silencing (Figure S2).We also investigated the changes in cell cycle distribution using Guava flow cytometer. As shown in Fig. 5b, downregulation of STAT6 in NCI-H460 cells caused no significant change in the distribution of population of cells in different phases of cell cycle.

The most significant network in the IPA analysis revealed genes like ATF5 *(Activating transcription factor 5)*, BCL2L1 *(BCL2-like 1)*, NUAK2 *(NUAK family, SNF1-like kinase, 2)* and TRIB3 *(Tribbles homolog 3)* which are directly or indirectly related to apoptosis. Our Real Time data showed that ATF5, BCL2L1, NUAK2 and TRIB3 decreased to 0.32, 0.57, 0.87 and 0.23 fold, respectively in siRNA transfected NCI-H460 cells in comparison to untransfected NCI-H460 cells (Fig. 5c). Simultaneously, we also checked for the change in expression of some pro-apoptotic and anti-apoptotic proteins by western blotting (Fig. 5d). We observed 0.85, 0.80 (p value  =  0.05) and 0.74 fold change in BCL-2 *(B-cell lymphoma 2)* levels, 0.96, 0.83 (p value  =  0.023) and 0.80 (p value  =  0.04) change in BCL-xL levels and there was slight increase in BAX *(BCL2-associated X protein)* levels with a fold change of 1.0, 1.37 (p value  =  0.05) and 1.1 at 24 h, 48 h and 72 h post siRNA transfection in NCI-H460 cells, respectively.

Additionally, we found that there was 0.87, 1.38 (p value  =  0.032) and 1.11 fold change in total TP53 *(Tumor protein p53)* levels, 1.2, 1.4 (p value  =  0.034) and 1.5 (p value  =  0.048) fold change in p-p53 *(phosphorylated p53)* levels, and 1.2, 1.3 (p value  =  0.05) and 1.6 fold (p value  =  0.046) change in PUMA (*p53 upregulated modulator of apoptosis*) levels upon STAT6 silencing at 24 h, 48 h and 72 h, respectively in NCI-H460 cells (Fig. 5d).

## Discussion

Signal transducer and activator of transcription-6 (STAT6) is a member of the STAT family of latent transcription factor and has been found to be overexpressed in various types of cancer like prostate and colon cancer [3], [28] http://www.ncbi.nlm.nih.gov/pubmed/18294957?dopt=AbstractPlus&holding=f1000,f1000m,isrctn. Several studies in the literature showed that down-regulation of STAT6 using siRNA leads to the induction of apoptosis [4], [29] but the underlying mechanism of STAT6 mediated signaling is not clear. To gain an understanding of the biological alterations we investigated the transcriptome before and after STAT6 silencing in NCI-H460 cells using illumina microarray.

Gene expression profiling and network analysis by IPA and PANTHER revealed *Gene expression, Cell death and Lipid metabolism* to be most favoured after STAT6-siRNA treatment in NCI-H460 cells. An important observation in the current study was the finding of cholesterol biosynthesis and p53 signalling in the toxicology list during IPA analysis. We observed significant increase in the cholesterol levels in NCI-H460 cells and in A549 cells as a result of STAT6 silencing (Fig. 3a). Our data also showed significant increase in the level of HMGCR, HMGCS1 and IDI1 (cholesterol synthesis) and INSIG1, and CYP27B1 (cholesterol homeostasis) genes at both transcriptional (real time PCR) and translational levels (western blot analysis). In this study for the first time we report that siRNA mediated silencing of STAT6 leads to the up-regulation of some of the genes involved in cholesterol biosynthesis/homeostasis resulting in enhanced cholesterol levels in the cells. Till date, there are only a few reports where indirect links between STAT6 and cholesterol biosynthesis have been observed. King *et al* in their study reported that IL-4 deficiency in C57BL/6 LDL receptor (LDLr)-/- mice promotes gallstone formation which maybe due to deregulation of genes involved in cholesterol metabolism, thereby implying that IL4 might be linked to cholesterol metabolism [30]. Black *et al* also observed that the use of IL-4 lowers the blood cholesterol levels [31]. We also found slightly decreased cholesterol levels after IL-4 treatment in our study. Herein, we show for the first time that direct inhibition of STAT6 can lead to an increase in cholesterol levels in lung cancer cells.

This inverse relationship of cholesterol biosynthesis and STAT6 is further strengthened by the reports on cholesterol lowering drugs simvastatin and atorvastatin which target HMGCR, lead to an increase in phosphorylated STAT6 [32], [33]. It is also well established that STAT6 is important for the development of asthma [34], [35], [36] and a study on US population by Fessler, *et al* in 2009 has shown that serum total cholesterol and non-HDL-cholesterol are inversely related to asthma [37], thereby further pointing towards the inverse relation between STAT6 and cholesterol.

In the present study, we not only observed inverse relationship of STAT6 and cholesterol biosynthesis but also found several conserved FOXJ2 and FOXD3 binding sites in the 5Kb region upstream of HMGCR, HMGCS1 and IDI1 (genes of the cholesterol synthesis pathway). FOXJ2 belongs to the family of forkhead box (FOX) proteins [38] and has been validated in our study using EMSA. [39]. Although, the structural and functional domains of FOXJ2 have been characterized [40], very little is known of the biological effects of FOXJ2 [41]. The enhanced binding of FOXJ2 to the key enzymes of cholesterol biosynthesis as a result of STAT6 silencing could have great potential in understanding the complexities of STAT6 signaling.

Several reports in the literature suggest that STAT6 knockdown is associated with inhibiting proliferation and enhancing apoptosis [1], [3]. Moreover, STAT6 has also been found to be activated spontaneously in human cancers such as prostate cancer [18], B cell lymphoma [42] and Hodgkin's lymphoma [43] suggesting that an active STAT6 signaling may be beneficial for cancer cell growth. In our study, cell death and p53 signaling came out as significantly altered upon STAT6 silencing as revealed by the IPA analysis (Fig. 2a and c). Since, p53 is well documented to have a role in apoptosis [44], [45] we checked the expression of the genes associated with p53 signaling such as p53, phosphorylated p53, PUMA, BCL-2, BAX, BCL-xL by western blot analysis. We observed increased expression of p53, phosphorylated p53, PUMA and BAX and reduced expression of BCL-2 and BCL-xL after STAT6 silencing. However, further study is needed to investigate the role of p53 in STAT6 signaling. Herein, we also observed an increase in annexin positive cells in a time dependent manner in control and STAT6 knockdown NCI-H460 cells. Contrary to our findings, an earlier study has shown that STAT6 induces apoptosis [46]. Our cell cycle analysis did not show any significant change in the number of cells in any phase. This is in contrast to the report by Zhang *et al* in 2005 where they had observed increased cells in G1 phase after STAT6 silencing in HT-29 cells [4]. Kaplan *et al* in 1998 had also reported that STAT6 deficient spleen cells show G1 arrest [47].

In conclusion, the study not only validates the novel finding of increased cholesterol levels after STAT6 silencing in NCI-H460 cells but also confirms the anti-apoptotic role played by STAT6. Since STAT6 can act as either pro-or antiapoptotic factor depending on the cell type a key pending question is whether STAT6 siRNA may have therapeutic activity in cancer. Future studies within the field of apoptosis will expand our understanding of the complex mechanisms underlying STAT6 mediated signalling. The knowledge of inverse relationship between STAT6 and cholesterol biosynthesis can be of significant potential in understanding and designing therapeutics for several pathological conditions where STAT6/cholesterol is implicated.

## Supporting Information

Figure S1 **Work flow of the illumina microarray experiment.**
(TIF)

Figure S2 **Effect of STAT6 silencing on apoptosis in A549 cells.**
(TIF)

Table S1 **5′-3′ Primer sequences used for Real-time PCR (FP- Forward Primer, RP-Reverse Primer).**
(XLS)

Table S2 **The list of 273 differentially expressed genes after transfection of STAT6 specific siRNA in NCI-H460 cells.**
(XLS)

Table S3 **The list of networks generated from IPA analysis using the differentially expressed genes obtained in the illumina array data.**
(XLS)

Table S4 **Genes in the most signifcant network identified by IPA with their fold change in the array data.**
(XLS)

Table S5 **Genes associated with the toxicology list generated from IPA analysis using the differentially expressed genes obtained in the illumina array data.**
(XLS)
