# Supplementary figures and images for: Small Interfering RNA against Transcription Factor STAT6 Leads to Increased Cholesterol Synthesis in Lung Cancer Cell Lines
Source: PLoS One. 2011 Dec 5;6(12):e28509. doi: 10.1371/journal.pone.0028509 (PMC3230611; doi:10.1371/journal.pone.0028509)

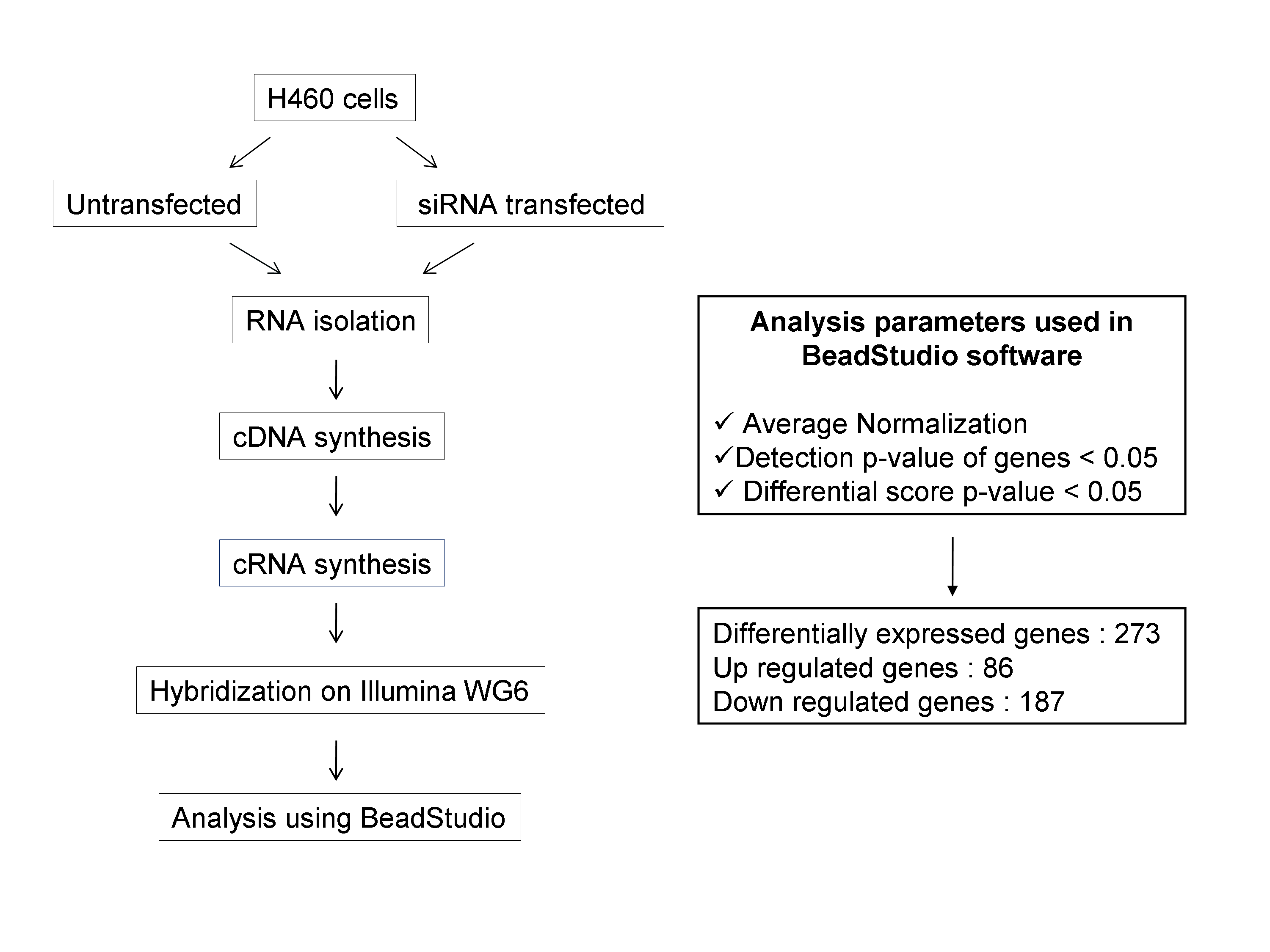

Supplement: Figure S1 — Work flow of the illumina microarray experiment. (TIF) [file pone.0028509.s001.tif]

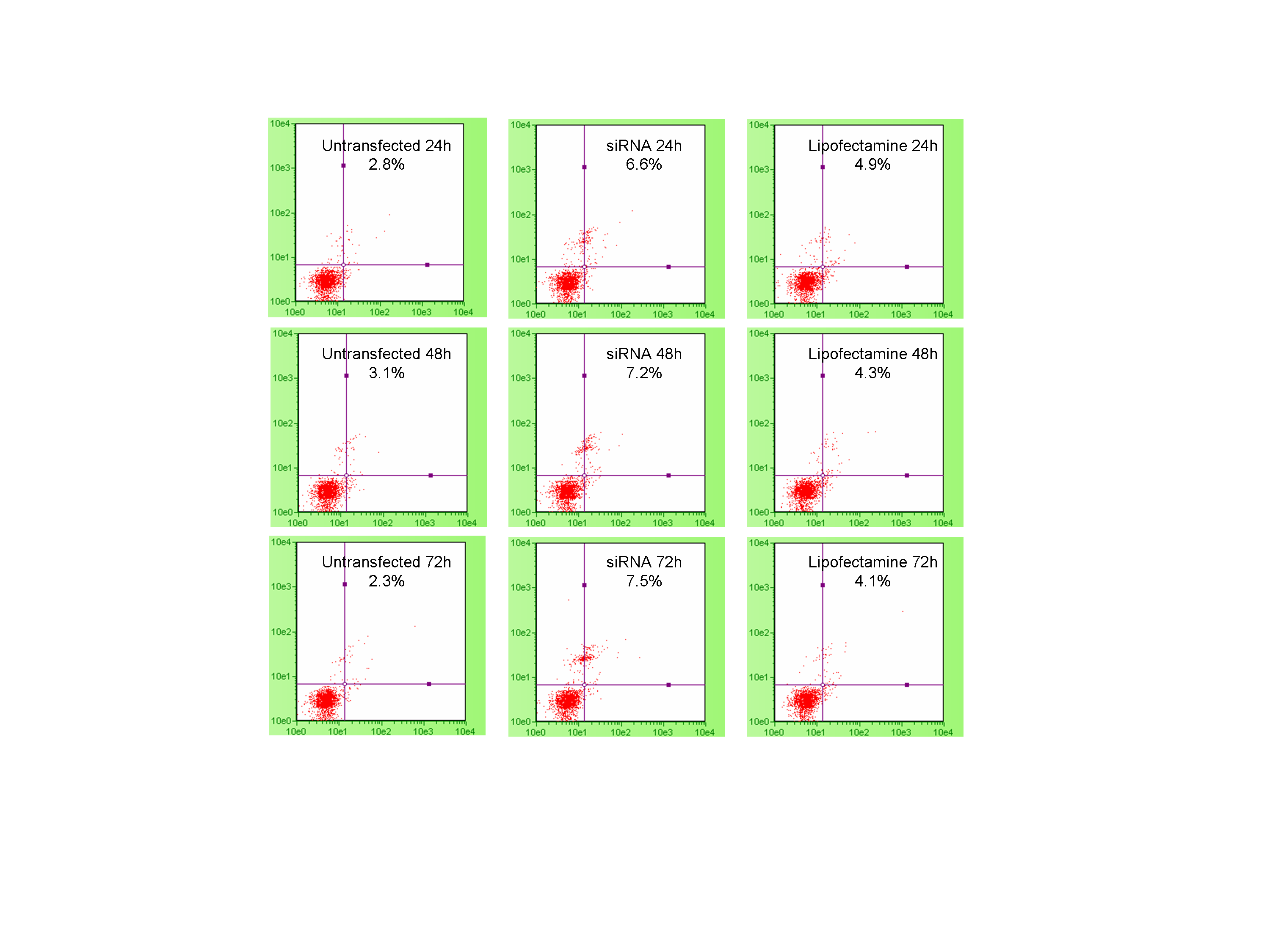

Supplement: Figure S2 — Effect of STAT6 silencing on apoptosis in A549 cells. (TIF) [file pone.0028509.s002.tif]
